# Short distance movement of genomic negative strands in a host and nonhost for *Sugarcane mosaic virus *(SCMV)

**DOI:** 10.1186/1743-422X-8-15

**Published:** 2011-01-13

**Authors:** Giovanni Chaves-Bedoya, Fulgencio Espejel, Ricardo I Alcalá-Briseño, Juan Hernández-Vela, Laura Silva-Rosales

**Affiliations:** 1 Laboratorio de Interacciones Planta-Virus. Departamento de Ingeniería Genética. Centro de Investigación y de Estudios Avanzados del IPN, CINVESTAV Irapuato, México; 2 Facultad de Ciencias Básicas e Ingenierías. Universidad de los Llanos. Villavicencio, Meta, Colombia

## Abstract

**Background:**

In order to obtain an initial and preliminary understanding of host and nonhost resistance in the initial step of potyvirus replication, both positive and negative *Sugarcane mosaic virus *(SCMV) strands where traced in inoculated and systemic leaves in host and nonhost resistant maize and sugarcane for one Mexican potyviral isolate (SCMV-VER1). Intermediary replication forms, such as the negative viral strand, seem to only move a short distance as surveyed by RT-PCR analysis and ELISA in different leaves. Virus purification was also done in leaves and stems.

**Results:**

Susceptible maize plants allowed for viral SCMV replication, cell-to-cell, and long distance movement, as indicated by the presence of the coat protein along the plant. In the host resistant maize plants for the SCMV-VER1 isolate, the virus was able to establish the disease though the initial steps of virus replication, as detected by the presence of negative strands, in the basal area of the inoculated leaves at six and twelve days post inoculation. The nonhost sugarcane for SCMV-VER1 and the host sugarcane for SCMV-CAM6 also allowed the initial steps of viral replication for the VER1 isolate in the local inoculated leaf. SCMV-VER1 virions could be extracted from stems of susceptible maize with higher titers than leaves.

**Conclusion:**

Nonhost and host resistance allow the initial steps of potyvirus SCMV replication, as shown by the negative strands' presence. Furthermore, both hosts allow the negative viral strands' local movement, but not their systemic spread through the stem. The presence of larger amounts of extractable virions from the stem (as compared to the leaves) in susceptible maize lines suggests their long distance movement as assembled particles. This will be the first report suggesting the long distance movement of a monocot potyvirus as a virion.

## Introduction

*Sugarcane mosaic virus *(SCMV) belongs to the genus *Potyvirus *within the family *Potyviridae *[1], which can infect different crops (e.g., sugarcane, sorghum, and maize) causing symptoms such as mosaics, chlorosis, and stunting [1] The SCMV is an important maize pathogen causing significant crop losses [2]. In Mexico, the *Sugarcane mosaic virus *was first reported in maize in 2006 [3]. Chemical control of the disease is not efficient due to the nonpersistent SCMV transmission by aphids [4]. The most efficient method of SCMV infection control is the cultivation of resistant maize varieties [5]

Host resistance is associated with dominant resistance, which in the case of maize relies on *Scmv*1 and *Scmv*2 genes [6]. Some resistance alleles are known to provide different levels of resistance depending on the host genome and virus, or pathotype [7]. Highly significant differences were found when studying genotype by environment interactions for resistance to SCMV in maize [8]. In terms of recessive genes, mutations in eIF4E and eIF(iso) 4E provide resistance to SCMV infections in different hosts [9,10] by disruption of their interaction with the VPg (Viral protein linked to the genome). This form of resistance can be caused by a defect in virus replication at the cellular level, a defect in cell-to-cell movement, or both. However, it is not always possible to differentiate between resistance affecting viral replication and accumulation at the cellular level, local, and long distance (systemic movement).

Nonhost resistance is normally described as resistance expressed by a plant species toward an specific pathogen and, compared to host resistance, is still very poorly understood [11]. Susceptibility, on the other hand, leads to a systemic infection when a virus is able to move, after genome amplification, from a primary site of infection, to distant parts of the plant. The infectious complex must move from cell-to-cell through plasmodesmata and long distances through the phloem [12].

Specialized movement proteins have not yet been described for potyviruses [13] like other viruses [14], but the coat protein (CP), helper component-proteinase (HC-Pro) and helicase (CI), seem to be necessary for the *Tobacco etch virus *(TEV) cell-to-cell and long distance movement [15-17]. Very little has been described on the involvement of proteins on the local and systemic translocation of the negative viral strand and even fewer dealing with its plant protein interactions [18].

The aim of this study was to make an initial attempt to characterize nonhost and host specific resistances to the Mexican isolate, SCMV-VER1, at the early stages of the virus replication. This was done comparing two maize lines (one susceptible, SL1 and one resistant RL1). In addition, two sugarcane lines CP-72-2086 and MY-44-12, nonhosts for the SCMV-VER1 isolate, were used. Evidence is provided toward a restriction in the long distance movement of genomic negative and positive strands in both types of resistant hosts.

## Results

**Figure 1:**
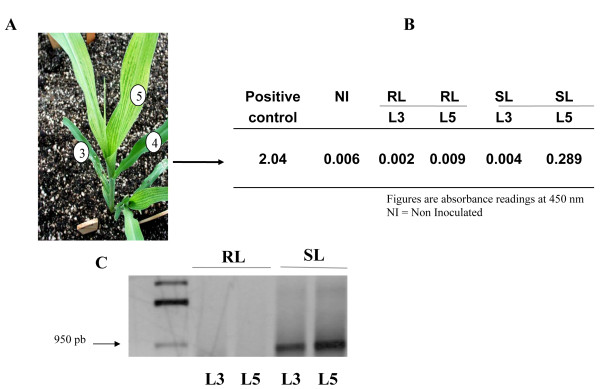
**Detection of SCMV in susceptible maize plants**. **(A) **The susceptible maize plant at five days post SCMV inoculation, showing the inoculated leaf (number 3), the systemic noninfected leaf (number 4), and systemic infected leaf (number 5). **(B) **The virus titers estimated by ELISA in either inoculated (L3) or systemic (L5) leaves in resistant (RL) and susceptible (SL) maize lines after SCMV inoculation. **(C) **RT-PCR amplification products of the SCMV coat protein (CP) cistron from inoculated leaves 3 and 5.

**Figure 2:**
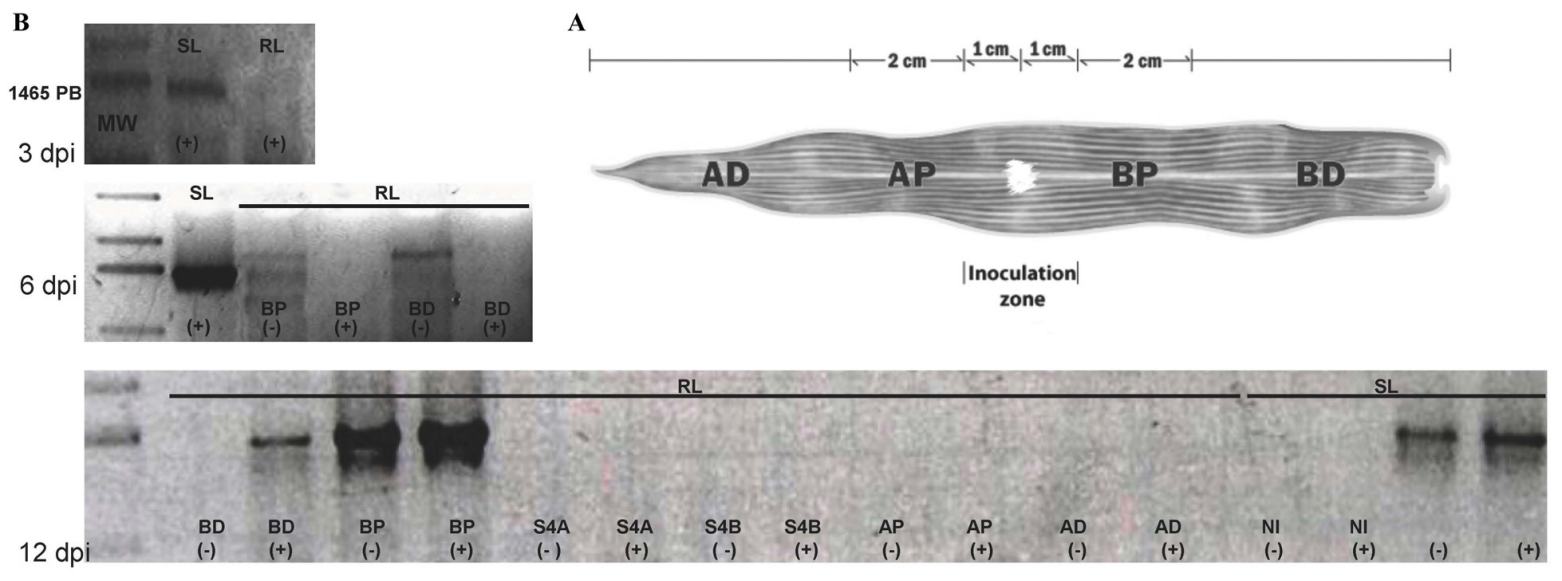
**Presence of the HC-Pro cistron in susceptible (SL) and resistant (RL) maize lines**. **(A) **The positions of the viral inoculated area and four relative positions from this site: two apical, one proximal (AP), and one distal (AD); and two basal, one proximal (BP), and one distal (BD), closer to the stem. **(B) **RT-PCR reactions showing either the presence or absence of the positive (+) or negative (-) amplified SCMV-HC-Pro cistron from the maize isolate (SCMV-VER1) at 3, 6, and 12 dpi in different positions, relative to the inoculation site. S4A and S4B are apical proximal and basal distal regions on a systemic leaf number 4. Noninoculated leaves are indicated as NI.

After SCMV-VER1 inoculation of the susceptible maize plants, mosaic symptoms were observed on number 5 leaves, but not on the number 3 inoculated leaves, nor on number 4 leaves, the subsequent systemic leaf (Figure 1A) in susceptible (SL) plants. Confirming these observations, ELISA detected the virus on number 5 leaves, but not on number 3 leaves in susceptible plants (Figure 1B). Neither symptoms nor virus were detected in the resistant maize line (RL) on any leaf along the plant at any time (Table 1). Although no virus was detected (by ELISA) on inoculated susceptible number 3 leaves, viral RNA corresponding to the CP cistron was present in this leaf, as shown by the amplified RT-PCR product (Figure 1C). This might either indicate that while viral RNA is present, not enough CP is present to be detected by ELISA, or that the RNA is a remnant of the virus inoculum. To investigate this, the inoculated leaf was divided in four sectors using the inoculation zone as a reference: two sectors in each the apical and basal halves. Each was subdivided into proximal and distal areas from the point of inoculation (Figure 2). RNA was then extracted from the different regions with the exception of the inoculation site; thus, the probability of residual RNA contaminating samples was kept at a minimum. No viral RNA (corresponding to the HC-Pro cistron) was detected in the RL plants in the inoculated leaf (number 3) three days post inoculation (3 dpi in Figure 2), indicating the lack of remnant RNA from inoculation, as well as the lack of viral replication in leaves from the resistant line.

**Table 1:** Viral detection by ELISA and RT-PCR for the CP of SCMV-VER1 infecting susceptible and resistant maize lines

		SL1 (susceptible)		RL1 (resistant)		
**dpi**	**Leaf no**.	**ELISA**	**RT-CPR**	**ELISA**	**RT-CPR**	**C+**

	3*	0.004	-	0.003	-	**1.002**
**1**	**4**	0.001	-	0.002	-	**1.304**
	**5**	ND*	ND*	ND*	ND*	ND*
	**3**	0.005	+	0.006	-	**1.001**
**3**	**4**	0.004	**+**	0.004	-	**1.111**
	**5**	ND*	ND*	ND*	ND*	ND*
	**3**	0.001	**+**	0.007	-	**1.405**
**6**	**4**	0.002	**+**	0.002	-	**1.204**
	**5**	**0.351**	**+**	0.004	-	**1.12**
	**3**	ND**	ND**	ND**	ND**	ND**
**9**	**4**	0.006	**+**	0.002	-	**0.985**
	**5**	**0.345**	**+**	0.012	-	**0.988**
	**3**	ND**	ND**	ND**	ND**	ND**
**12**	**4**	0.010	**+**	0.015	-	**1.203**
	**5**	**0.387**	**+**	0.020	-	**1.145**

ND* = Not determined/not formed leaf

ND** = Not determined/senescing leaf

**Figure 3:**
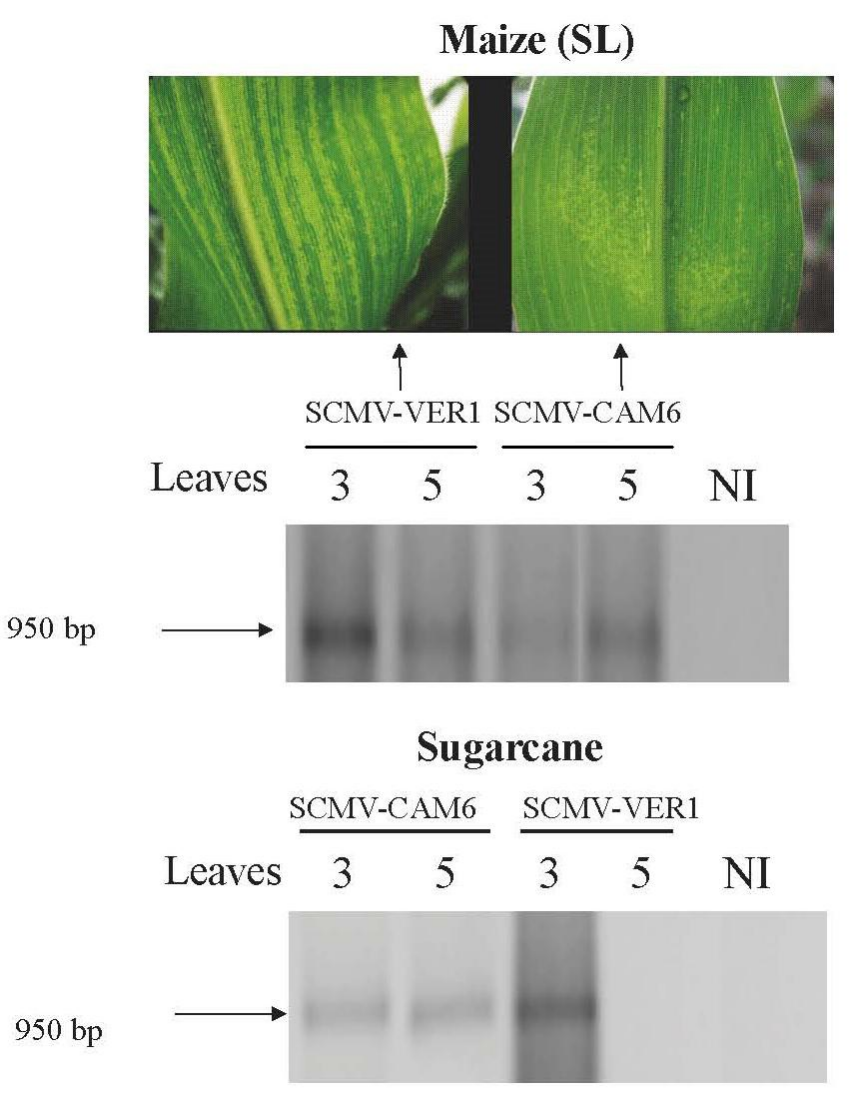
**Host and nonhost SCMV symptoms and replication**. RT-PCR reactions showing either the presence or absence of the negative amplified strand of SCMV-CP cistron from the maize (VER1) and sugarcane (CAM6) isolate on the inoculated or systemic leaves (L3 and L5). NI refers to noninoculated leaves.

In order to understand if the lack of viral RNA in this zone would be a reflection of resistance at replication level, the negative genomic strand was traced and detected exclusively in the inoculated leaf's basal area (either BD or BP) at 6 and 12 dpi in the RL line (Figure 2). To determine whether this type of response would be similar in a nonhost plant, like the one presumed in sugarcane plants (hosts for SCMV-CAM6, but not for SCMV-VER1 isolate), both species (maize and sugarcane) were each inoculated with the SCMV-CAM6 and SCMV-VER1 isolates. SCMV-CAM6 produced mild symptoms in susceptible (SL) systemic maize leaves (Figure 3). The negative SCMV-CAM6 strand was present on both maize and sugarcane inoculated and systemic leaves (numbers 3 and 5, respectively) (Figure 3). On the other hand, the sugarcane plants developed systemic symptoms when inoculated with SCMV-CAM6, but none with the SCMV-VER1. The negative SCMV-VER1 band was detected only in the inoculated sugarcane leaf (basal area of leaf 3) (Figure 3 lower panel).

**Figure 4:**
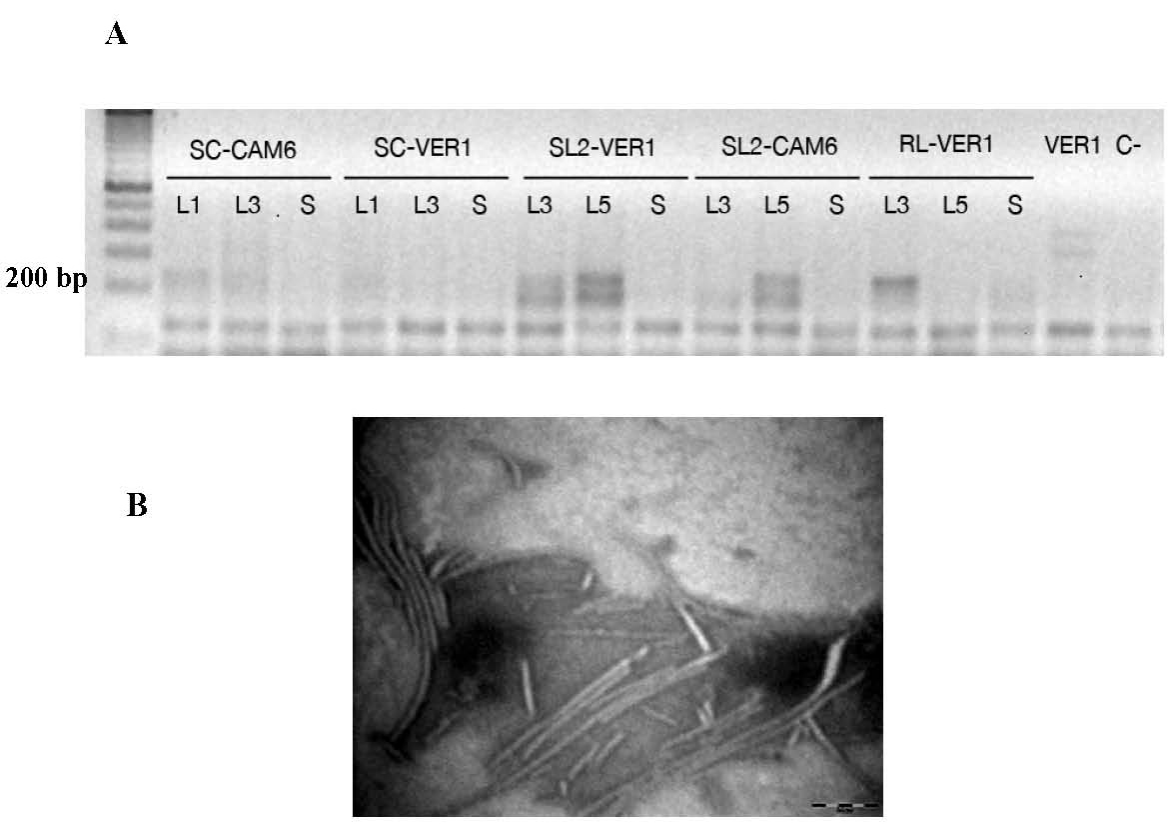
**Negative strands short distance movement of P1/HC-Pro cistrons and virions long distance movement**. **(A) **RT-PCR reactions showing either the presence or absence of the negative amplified strands of SCMV-VER1 and SCMV-CAM6 in maize (L3 and L5) and sugarcane (L1 and L3), in the inoculated and systemic leaves, respectively. SCMV-susceptible (SL2) maize lines were tested, as well as a maize host (RL) and the sugarcane (SC) nonhost resistant to SCMV-VER1. **(B) **Electron micrographs of SCMV virions extracted from the stem of susceptible maize lines showing the expected size and aspect. The particles were negatively stained with 3% PTA pH 6.89. Bar = 2 um.

In order to assess whether the negative strand would move toward the stem, a search for genomic negative strands was carried out at 10 dpi when the systemic infection would have been place and symptoms in the majority of the leaves. Leaves 3 and 5, and their corresponding stem sections, were surveyed (Figure 4A). A degenerate primer was then used that would amplify genomic regions within the HC-Pro and P1 cistrons. There was a lack of negative genomic strands (or very faint bands) on all stems (S): the sugarcane, host for SCMV-CAM6 and the nonhost for SCMV-VER1, the two susceptible maize lines (SL) SCMV-CAM6, and the resistant maize line (RL) for SCMV-VER1. As expected, viral negative strands were present in the host susceptible lines (SL2 for SCMV-CAM6 and -VER1, respectively), as well as in the inoculated RL maize leaf. Since no negative genomic strands were present in the stem, a search for virions was performed through viral purification of the leaves and stems, separately. Three different viral purifications were performed in the stems and leaves of SL 1 infected plants. Average yields of the pure virus per 100 g of tissue were 60 μg for leaves and 100 μg for stems, thus suggesting more assembled particles moving longer distances along the stem than short distances in the leaves. The viral integrity of particles was assessed in both cases through TEM (transmission electron microscopy) images from viral purifications revealing potyvirus particles and thus supporting the evidence of SCMV as assembled virions in the stem of SL maize (Figure 4B). No viral particles could be purified from infected RL stems.

In order to confirm the nature of the stem-purified particles, a protein analysis was conducted using mass spectrometry after excision of the corresponding SDS-PAGE band from the purified particles. Mascot http://www.matrixscience.com results indicate that the 40 kDa protein detected from the excised band corresponds to the amino acid composition of the CP of SCMV.

## Discussion

In this study, we report initial results on the presence and short distance movement of intermediary replication viral strands, i.e., negative genomic strands, in a host resistant maize line (RL1), as well as in a nonhost (sugarcane) for the SCMV-VER1 isolate. Reports of SCMV local movement in resistant germplasms have previously been reported [19,20] by either monitoring the presence of the CP or immunofluorescent staining of the viral infection.

Virus movement in plants is thought occur through cell-to-cell and systemic movement through the phloem [21], mainly as RNA-movement protein complexes [14]. For potyviruses, CP protein is considered as an important factor in short and long distance movement [17], similar to TMV. It has been shown that some TMV mutants are repressed in systemic movement, but not for local spread [22]. This observation indicates that the phloem import mechanism is different of cell-to-cell movement in potyvirus. We present evidence of the presence of SCMV virions in maize stems. This suggests that SCMV is capable of long distance spread in monocot maize SL plants as virions, as previously showed for other viruses (e.g., the *Cucumber mosaic virus*) using electron microscopy in sieve elements of *Nicotiana *plants [23].

Our results also show that the SCMV-VER1 isolate is able to go through the initial steps of replication in host resistant maize (RL) and nonhost sugarcane (SC), and can be detected in its replicative form at 5 dpi (Figure 3). This suggests the existence of uncoated viral RNAs in the replication complexes. Considerably shorter times for uncoating (e.g, 45 seconds) have been suggested for other non potyvirus examples, such as the *Turnip yellow mosaic virus *(TYMV), a *Tymovirus*, [24], or hours for TMV [25].

SCMV negative strand detection by RT-PCR in resistant maize plants was possible at 6 dpi but no earlier. Both positive and negative intermediate replication RNA strands were detected in the basal proximal region from the inoculation site, suggesting that in this zone, the virus can replicate and have a short distance movement as it does in susceptible maize plants. The direction of the negative strands' movement is suggestive of viruses' well-documented movement along the photoassimilate pathway from a source to a sink leaf [26]. At 12 dpi, both positive and negative RNA forms were also found in the basal regions of the resistant maize plants. At this time, we were unable to identify strands of either polarity in systemic leaves, suggesting a restriction of the virus long distance movement. TMV demonstrated the ability to move from cell-to-cell, from the initial inoculation spot through the plasmodesmata [27], go through the initial replication step and move as a large replication complex inside the cell through the use of microfilaments, and then between adjacent cells [25]. Potyviral replication complexes have also been observed to move as intracellular vesicles containing replication components [28,29]. A similar SCMV complex could move a short distance in the inoculated leaf, down to its basal area, and near it insertion to the stem. The presence of negative strands would agree with the replication complex movements proposed for TuMV [28] and TMV [25]. The viral replicase, RdRp, would replicate the viral genomic RNA if present, in the vesicles, producing the negative strands along its moving path. Inside these moving vesicles, a disrupted interaction between the possible eIF(iso)4E, eIF4E, or eIF4G from recessive resistant genes and the viral VPg could still take place, resulting in resistance towards SCMV similar to other resistant genes for potyviruses [30,31] and not allowing its translation; hence, the lack of viral proteins (Figure 1B). Nevertheless, such vesicles process of long distance movement along either the phloem sieve tubes or companion cells need to be studied in the host and nonhost resistant genotypes. Long distance movement as a ribonucleotide complex has been suggested by prior research [14,32-36]. However, further research is needed regarding such vesicles' participation in the long distance movement. Movement as viral particles has been reported for viruses such as the *Cucumber mosaic virus *[37] and the *Cucumber green mottle virus *[38].

In maize plants, host proteins should be looked at in important anatomical structures connecting leaf blades and stems, such as the ligules [39], where the transition from replicating genomic RNA complexes to virions that are ready to be transported long distances are likely to take place. The search for proteins that are reportedly involved in these stems and structures' systemic movement will be done in both the non- and host plants for SCMV-VER1.

## Conclusion

In resistant host maize and nonhost sugarcane plants, the first step in viral replication and movement of replication intermediaries for SCMV is allowed, but the long distance movement in susceptible plants seems to occur as assembled virions through the plant vasculature.

## Materials and methods

### Plant inoculation

The SCMV maize isolate (SCMV-VER1 Accession no. EU091075) was obtained from an infected plant in Poza Rica, Veracruz in Mexico [3]. The SCMV sugarcane isolate from Cameroon was used as a reference isolate (SCMV-CAM6-1), and was donated by M. Peterschmitt from CIRAD, France. The susceptible CIMMYT SL1 and DAS 2348 (SL2), and the resistant CIMMYT RL1 were the maize germplasms used in this work, kindly transferred by that institution. Sugarcane plants used were from the ° lines. Mechanical virus inoculation was done in either the sugarcane's third leaf or in the maize plants at the four-leaf growth stage (ca. 15 days after sowing). Leaf 1 refers to the oldest basal leaf in the plant. Mock and virus inoculated plants were grown under greenhouse and growth chamber conditions (24°C, 16 hr light, and 200 μE). Virus inocula were prepared by grinding 100 mg of young, infected leaf tissue in 1 ml of inoculation buffer (10 mM phosphate buffer, pH 7). A pure virus was also used to inoculate maize plants. Virus purification was done as previously described [40]. Mechanical inoculation was achieved using carborundum as an abrasive, or if the pure virus was used, a syringe was injected in the leaf's abaxial surface. Leaf samples were collected at 2, 4, 6, 9, and 12 dpi (days post inoculation), either using total leaves or parts of them, at different zones away from the inoculated, one square centimeter spot, and referred to as: BP (basal proximal), BD (basal distal), AP (apical proximal), AD (apical distal), S4BD (systemic leaf number 4, BD), and S4AP (systemic leaf number 4, AP). Whole leaves, or samples from each leaf zone not larger than two centimeters by the width of the leaf (Figure 1), were used in ELISA and/or detection of the positive and negative viral genome strands. Mock buffer inoculated plants were used as negative controls.

### Virus detection by ELISA

The double-antibody sandwich indirect method of the enzyme-linked immunosorbent assay (DAS-ELISA) was performed using a commercial SCMV kit (Agdia, PathoScreen, Elkhart, IN). For this test, either the purified virus or 100 μl of crude leaf extracts (in 1:10 w/v extraction buffer) were used. The color reaction was developed using p-nitrophenyl phosphate (PNP), and the absorbance was read at 405 nm in a microtiter plate reader (Ultramark Bio-Rad).

### RT-PCR reactions for positive and negative strand detection

Total RNA was extracted with the Trizol reagent according to the manufacturer's instructions from the different indicated leaf zones. After quantification, the total RNA was used as a template to amplify the positive and negative genomic strands depending on the primer used in the reverse transcription: the reverse oligonucleotide (3'primer), for the amplification of the positive strand, and the forward primer (5'primer) for the negative one. Primers were directed against either the coat protein (CP) or the helper component-proteinase (HC-Pro) cistrons, as indicated in each Figure. For the HC-pro cistron, the forward primer sequence was 5' - TCGTGCGTGGAAGGATGC -3', and the reverse primer sequence was 5'-GAGATAAGCACGGTAGGG-3'. The size of the expected PCR for the HC-Pro is 1582 bp. For the CP cistron, the forward primer sequence was 5'-TCCGGAACTGTTGATGCGGGTGTACAAG-3', and the reverse primer sequence was 5'- CTAGTGGTGATGCTGCACTCCCAACAGA-3'. The size of the expected PCR for the CP is 950 bp. Degenerate primers were also used to detect P1, HC-Pro, and NIb cistrons in the stem assays. PCR conditions on the RT products were: one cycle at 94°C, 30 cycles each of 30 sec at 94°C; 35 sec at 52°, or 59 °C (for HC-Pro and CP, respectively); 2 min at 72°C, and one final cycle of 7 min at 72°C. Amplified products were run on either a 0.8% or 2% agarose gel and photographed for analysis after ethidium bromide or GelRed ™ exposure for DNA staining. The primer design was based on the SCMV-VER1 sequence described above.

### Virus purification

SCMV purification was done according to the protocol on MDMV (maize dwarf mosaic virus) [40]. 100 to 200 g of symptomatic leaves (or stems) from infected plants were used as the starting material and ground in a blender with carbon tetrachloride 5% (v/v) and 0.25% Triton-X100 for a 10 min centrifugation at 15 000 g at 4 °C. This was followed by 6% PEG precipitation and pellet resuspension on 0.1 M of ammonium citrate pH6 with 1% polyvinyl pyrrolidone, and 0.5% 2-mercaptoethanol and a 10 min centrifugation at 10 000 g. The next steps were a 90 min 100 000 g centrifugation on a sucrose 20% pad, a final passage in a 10-40% sucrose density gradient for 2 h at 100 000 g, and dialysis on the ammonium resuspension buffer without 2-mercaptoethanol. Absorbance readings were done at 260-280 nm for yield estimations. A total of four virus purifications were conducted.

### SDS-PAGE

Viral purification from susceptible maize stems was used to run a 12% polyacrylamide gel in a Laemmli buffer. Electrophoresis was run at 4°C and at a constant voltage. The gel was further stained with Coomassie brilliant blue.

### Analysis of proteins by mass spectrometry

Protein analysis using mass spectrometry (MS) was conducted, as described previously [41]. In brief, the band of interest (approximately 40 KDa) was cut off from the SDS-PAGE. The excised fragment from the gel was washed with water, and then with 50% (v/v) acetonitrile in water, acetonitrile mixed with 100 mM of ammonium bicarbonate (1:1), and 100% acetonitrile. Protein in the gel was digested with trypsin (using sequence grade trypsin from PROMEGA), and the resulting peptides were extracted. The peptides obtained were analyzed with a matrix-assisted laser desorption ionization-time of flight mass spectrometry (MALDI-ToF MS) using an Ettan MALDI-ToF Pro instrument. The experimental mass values were compared with those derived from available databases using the Mascot program.

### Transmission Electron Microscopy (TEM)

TEM experiments to detect viral particles in the maize stem were conducted, as previously described ([42] using standard negative staining techniques.

## Competing interests

The authors declare that they have no competing interests.

## Authors' contributions

GCB conducted part of the molecular work and helped to write the paper. FE participated in the design the study, and assisted with the molecular work and genetic analysis. RIA-B also contributed to the molecular work. JHV did viral extractions and microscopic analyses. LS-R conceived of the study, drafted, and wrote most of the paper. All authors read and approved the final manuscript.
